# Extensive cerebral venous thrombosis associated with severe hyperhomocysteinemia in a child: a case report

**DOI:** 10.11604/pamj.2026.53.55.51203

**Published:** 2026-02-04

**Authors:** Li Han, Qian Li, Guangxiang Chen

**Affiliations:** 1 Department of Radiology, The Affiliated Hospital, Southwest Medical University, Sichuan, China,; 2 Department of Radiology, The Fourth People's Hospital of Zigong, Zigong, China

**Keywords:** Cerebral tenous thrombosis, hyperhomocysteinemia, ectopia lentis, thrombectomy, case report

## Abstract

Pediatric cerebral venous thrombosis (CVT) is a rare and life-threatening condition, and hyperhomocysteinemia serves as a significant risk factor. We report an 11-year-old female presenting with blurred vision, gait instability, nausea, vomiting, and a persistent one-week headache. Her medical history of congenital ectopia lentis and prior bone fracture offered a pivotal diagnostic lead. Brain magnetic resonance imaging (MRI) confirmed extensive dural sinus thrombosis, and laboratory testing revealed severe hyperhomocysteinemia. The therapeutic regimen included anticoagulation, vitamin B6 supplementation, and thrombectomy of the venous sinus. After 15 days of treatment, the patient demonstrated significant amelioration of headache and motor deficits. Therefore, monitoring serum homocysteine levels is crucial in the management of CVT to ensure a comprehensive assessment of the patient's condition and therapeutic efficacy.

## Introduction

Severe hyperhomocysteinemia is a well-established risk factor for atherosclerosis and ischemic stroke [1]. An increase in homocysteine and a deficiency of cysteine in patients with homocystinuria may impair normal zonular fiber development, which may lead to complete dislocations or lens subluxations [2]. We report a patient with a known history of lens dislocation who had not undergone prior metabolic screening; the diagnosis of hyperhomocysteinemia was established only after the formation of extensive cerebral venous thrombosis (CVT). This case underscores the critical importance of serum homocysteine testing. Particularly in adolescents presenting with lens dislocation or cerebral venous thrombosis, clinicians must monitor homocysteine levels to ensure a comprehensive assessment of the patient's condition and therapeutic efficacy.

## Patient and observation

**Patient information:** an 11-year-old female presented to the pediatric emergency department with a chief complaint of blurred vision, gait instability, nausea, vomiting, and a persistent headache lasting for one week. Her past medical history was significant for congenital ectopia lentis requiring intraocular lens implantation surgery five years ago, premature graying of hair noted two years ago, and a left radial fracture sustained five months ago following a fall. She denied any history of head trauma, febrile illness, or known allergies, and there was no family history of venous thromboembolism.

**Clinical findings:** physical examination was notable for left-sided hemiparesis, with decreased muscle strength in the upper and lower limbs. Additionally, premature graying of the hair was observed. Examination of the left forearm revealed no obvious deformity or tenderness at the site of the previous radial fracture.

**Figure 1:**
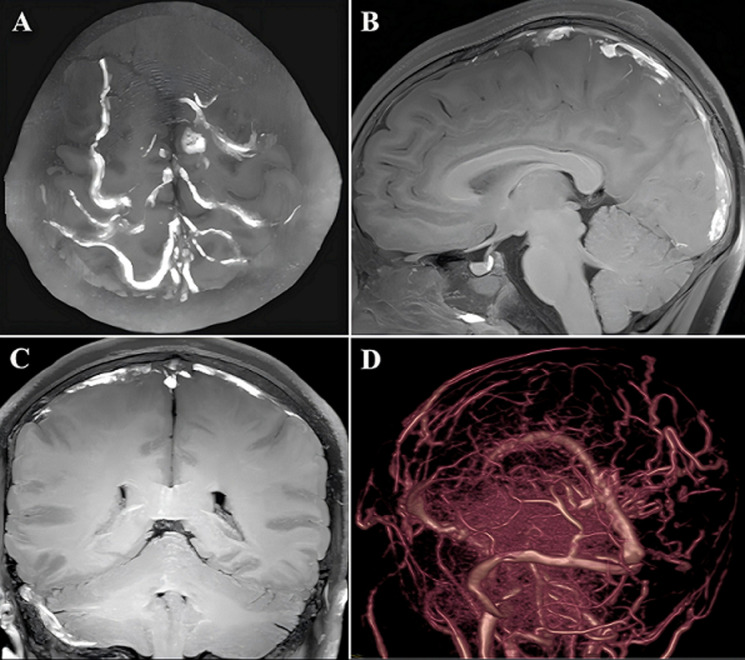
brain magnetic resonance imaging of the patient; A,B,C) maximum intensity projection from 3D-T1W high-resolution, magnetic resonance imaging revealed extensive cerebral venous thrombosis in the right sigmoid sinus, transverse sinus, superior sagittal sinus and multiple venous branches on the surface of the cerebrum; D) magnetic resonance venography revealed that the above venous sinuses were not visualized, and plenty of collateral vessels were visible

**Diagnostic assessment:** laboratory testing revealed a markedly elevated serum homocysteine concentration of 114.5 µmol/L (reference range: 6-14 µmol/L). High-resolution 3D-T1W MRI of the brain confirmed extensive cerebral venous thrombosis (Figure 1A-C) and prominent collateral circulation (Figure 1D). Collectively, the characteristic triad of ectopia lentis, skeletal fragility, and thrombosis, combined with severe hyperhomocysteinemia, indicates a high probability of homocystinuria. The patient´s neurological condition was identified as extensive cerebral venous thrombosis secondary to hyperhomocysteinemia. However, due to the emergency presentation and critical condition of the patient, a genetic diagnosis for homocystinuria could not be obtained in a timely manner.

**Therapeutic interventions:** upon admission, we initiated systemic anticoagulation and vitamin B6 supplementation. Mannitol was administered to control intracranial pressure, alongside supportive care to maintain homeostasis. Given the patient's significant neurological impairment and critical condition, a multidisciplinary team (MDT) consultation was held, which confirmed the indication for endovascular therapy. Subsequently, the patient underwent cerebral venography, mechanical thrombectomy, balloon angioplasty, and venous sinus stenting.

**Follow-up and outcome of interventions:** the patient's postoperative recovery was uneventful. By day 15, her headache and hemiparesis had significantly improved, and the neurological examination was unremarkable, allowing for discharge. At the 4-month follow-up, she had successfully returned to her normal daily activities. Repeat MRI and magnetic resonance venography (MRV) demonstrated good visualization of the cerebral veins with no evidence of new cerebral infarction or thrombosis. Additionally, her serum homocysteine level had decreased to 75.3 µmol/L. The patient was advised to strictly adhere to a schedule of regular follow-up for coagulation profiling, cranial MRV, and homocysteine monitoring.

**Patient perspective:** the patient and her family expressed satisfaction with the diagnostic and treatment process, acknowledging the therapeutic efficacy. She specifically reported a significant improvement in her headache and hemiparesis.

**Informed consent:** written informed consent was obtained from the patient´s father for publication of this case report and any accompanying images.

## Discussion

Homocystinuria is a congenital disorder of sulfur-containing amino acid metabolism, primarily caused by a deficiency in cystathionine beta-synthase (CBS). The major clinical manifestations include intellectual disability, seizures, ectopia lentis, skeletal deformities, and occlusive vascular disease [1]. Risk factor analysis for cerebral venous sinus thrombosis indicates that hyperhomocysteinemia is a primary risk factor in up to 22.3% of the male population [3]. Although our patient presented without evidence of cephalofacial or systemic infection or coagulation abnormalities, she exhibited significant hyperhomocysteinemia. Homocysteine, an intermediate metabolite of methionine and cysteine, has been confirmed as a risk factor for atherosclerosis and ischemic stroke, as it induces a hypercoagulable state that precipitates cerebral venous sinus thrombosis (CVST). Furthermore, the zonules of Zinn (suspensory ligaments of the lens) are rich in cysteine, which functions to maintain the normal position of the lens. In patients with homocystinuria, elevated homocysteine levels combined with cysteine deficiency may impair the normal development of these zonular fibers, leading to lens subluxation or complete dislocation [2,4]. Therefore, long-term exposure to high serum homocysteine levels offers the most plausible explanation for the bilateral ectopia lentis observed in this patient.

The gold standard for diagnosing homocystinuria is genetic sequencing, yet this is often not immediately available in the emergency setting. In the present case, the constellation of ectopia lentis, a history of fractures, and extensive cerebral venous thrombosis provided critical diagnostic clues, which were subsequently corroborated by markedly elevated serum homocysteine levels. Given that pediatric CVT is a rare but life-threatening cerebrovascular event, comprehensive physical examination, biochemical analysis, and neuroimaging are of utmost importance for pediatric patients presenting with acute cerebrovascular symptoms. Timely diagnosis and aggressive intervention are of paramount clinical importance. While the management of pediatric CVT primarily relies on systemic anticoagulation, endovascular therapy (EVT) is typically reserved for patients exhibiting rapid neurological deterioration or refractory thrombosis. In our patient, the presence of extensive cerebral venous thrombosis accompanied by hemiparesis indicated significant neurological impairment. Consequently, following an MDT consultation, we proceeded with venous thrombectomy and venous sinus stenting to facilitate rapid neurological recovery. The favorable outcome in this case suggests that for complex pediatric CVT, particularly when the underlying hypercoagulable state (such as hyperhomocysteinemia) cannot be rapidly reversed, endovascular stenting serves as a viable and effective salvage therapy. During follow-up, we observed that while the patient´s neurological symptoms significantly improved with no new cerebral venous thrombosis, her serum homocysteine levels have not yet fully returned to the normal range. Consequently, regular re-evaluation and follow-up are of particular importance.

## Conclusion

Hyperhomocysteinemia is a significant risk factor for cerebral venous thrombosis (CVT). Therefore, routine screening of serum homocysteine levels is essential for the comprehensive evaluation and management of CVT, particularly in children and adolescents. Monitoring these levels is critical for assessing therapeutic efficacy, and active intervention holds significant clinical importance.
